# *catena*-Poly[hexaaqua-1κ*O*,2κ*O*,3κ^4^ *O*-(μ_4_-3,5-dicarboxylatopyrazol-1-ido-3′:1:2:3κ^6^ *O* ^5^:*N* ^1^,*O* ^5′^:*N* ^2^,*O* ^3^:*O* ^3′^)(μ_2_-3,5-dicarboxylatopyrazol-1-ido-1:2κ^4^ *N* ^2^,*O* ^3^:*N* ^1^,*O* ^5^)-1,2-dicopper(II)-3-manganese(II)]

**DOI:** 10.1107/S1600536810037542

**Published:** 2010-09-30

**Authors:** Xin-Hui Zhou

**Affiliations:** a Key Laboratory for Organic Electronics & Information Displays (KLOEID), and Institute of Advanced Materials (IAM), Nanjing University of Posts & Telecommunications (NUPT), Nanjing 210046, People’s Republic of China

## Abstract

In the title compound, [Cu_2_Mn(C_5_HN_2_O_4_)_2_(H_2_O)_6_]_*n*_, the Cu^II^ ion is coordinated by two N atoms, two O atoms and one water O atom in a distorted square-pyramidal geometry. The Mn^II^ ion is coordinated by two O atoms and four water O atoms in a distorted octa­hedral geometry. Two pyrazolyl-3,5-dicarboxyl­ate anions chelate to two copper ions, forming a dinuclear unit, which further connects the Mn^II ^ions into chains extending along [100]. Both independent coordinated water mol­ecules on the Mn^II^ ion are disordered in a 50:50 fashion.

## Related literature

Pyrazole-3,5-dicarb­oxy­lic acid is a multifunctional ligand which exhibits versatile coordination modes, see: Pan *et al.* (2001 ▶); Zhou *et al.* (2009 ▶). For related structures, see: King *et al.* (2004 ▶). 
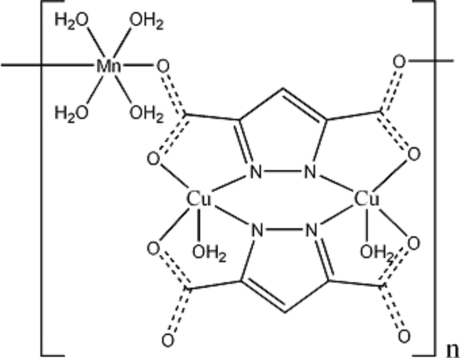


## Experimental

### 

#### Crystal data

[Cu_2_Mn(C_5_HN_2_O_4_)_2_(H_2_O)_6_]
*M* *_r_* = 596.27
Orthorhombic, 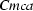

*a* = 21.778 (3) Å
*b* = 13.0387 (19) Å
*c* = 12.3800 (18) Å
*V* = 3515.3 (9) Å^3^
*Z* = 8
Mo *K*α radiation
μ = 3.19 mm^−1^
*T* = 291 K
0.15 × 0.14 × 0.12 mm

#### Data collection

Bruker SMART APEX CCD diffractometer
Absorption correction: multi-scan (*SADABS*; Bruker, 2000 ▶) *T*_min_ = 0.646, *T*_max_ = 0.700
8862 measured reflections
1779 independent reflections
1562 reflections with *I* > 2σ(*I*)
*R*_int_ = 0.058

#### Refinement

*R*[*F* ^2^ > 2σ(*F* ^2^)] = 0.057
*wR*(*F* ^2^) = 0.126
*S* = 1.26
1779 reflections
164 parameters
H-atom parameters constrained
Δρ_max_ = 0.64 e Å^−3^
Δρ_min_ = −0.64 e Å^−3^

### 

Data collection: *SMART* (Bruker, 2000 ▶); cell refinement: *SAINT* (Bruker, 2000 ▶); data reduction: *SAINT*; program(s) used to solve structure: *SHELXTL* (Sheldrick, 2008 ▶); program(s) used to refine structure: *SHELXTL*; molecular graphics: *SHELXTL*; software used to prepare material for publication: *SHELXTL*.

## Supplementary Material

Crystal structure: contains datablocks I, global. DOI: 10.1107/S1600536810037542/fj2333sup1.cif

Structure factors: contains datablocks I. DOI: 10.1107/S1600536810037542/fj2333Isup2.hkl

Additional supplementary materials:  crystallographic information; 3D view; checkCIF report

## Figures and Tables

**Fig. 1.**
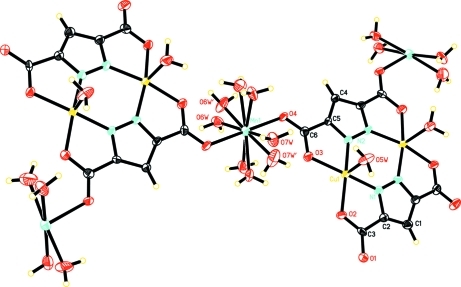
Crystal structure of (I) with labeling and displacement ellipsoids drawn at the 50% probability level.

## supplementary crystallographic information

### Comment

Pyrazole-3,5-dicarboxylic acid is a multifunctional ligand and exhibits the versatile coordination modes (Pan *et al.* 2001; Zhou *et al.* 2009). As a part of our ongoing investigations in this ligand, we report here the crystal structure of the title compound. In the crystal structure of the title compound, the Cu atom is coordinated by two carboxylate oxygen atoms and two pyrazolyl nitrogen atoms from two pyrazolyl-3,5-dicarboxylate anions and one water molecule in a distorted square-pyramidal geometry. The Mn atom is coordinated by two O atoms from two pyrazolyl-3,5-dicarboxylate anions and four water molecules in a distorted octahedral geometry (Figure 1). Each two pyrazolyl-3,5-dicarboxylate anoins chelate to two copper ions to form the dinuclear Cu(II) unit with the Cu···Cu distance of 3.883 (1) Å. These dinuclear Cu(II) units connect the manganese atoms by two remaining carboxylate oxygen atoms from a pyrazolyl-3,5-dicarboxylate anion into chains that elongate in the direction of the crystallographic *a* axis.

### Experimental

A mixture of pyrazole-3,5-dicarboxylic acid (0.2 mmol, 34.8 mg), CuI (0.1 mmol, 19 mg), MnCl_2_.4H_2_O (0.1 mmol, 19.8 mg), KI (0.1 mmol, 16.6 mg), CH_3_CN (4 ml) and H_2_O (2 ml) was sealed in a 15 ml Teflon-lined bomb and heated at 140°C for 3 days. The reaction mixture was slowly cooled to room temperature to obtain the blue block crystals of (I) suitable for X-ray diffraction analysis.

### Refinement

H atoms were placed in calculated positions with C—H = 0.93 Å, and refined in riding mode with *U*_iso_(H) = 1.2*U*_eq_(C). The H atoms of the water molecules were located in difference map, their bond lengths were set to 0.85 Å and afterwards they were refined using a riding model with *U*_iso_(H) = 1.5*U*_eq_(O).

The atom O6 is disordered in two positions, with site occupancy factors of 0.50 (3) and 0.50 (3). The atom O7 is disordered in two positions, with site occupancy factors of 0.50 (2) and 0.50 (2). The total occupancy sums of both positions for O6 and O7 are 1.

### Crystal data

**Table:**

[Cu_2_Mn(C_5_HN_2_O_4_)_2_(H_2_O)_6_]	*F*(000) = 2376
*M**_r_* = 596.27	*D*_x_ = 2.253 Mg m^−^^3^
Orthorhombic, *C**m**c**a*	Mo *K*α radiation, λ = 0.71073 Å
Hall symbol: -C 2bc 2	Cell parameters from 2617 reflections
*a* = 21.778 (3) Å	θ = 2.5–28.3°
*b* = 13.0387 (19) Å	µ = 3.19 mm^−^^1^
*c* = 12.3800 (18) Å	*T* = 291 K
*V* = 3515.3 (9) Å^3^	Block, blue
*Z* = 8	0.15 × 0.14 × 0.12 mm

### Data collection

**Table:**

Bruker SMART APEX CCD diffractometer	1779 independent reflections
Radiation source: fine-focus sealed tube	1562 reflections with *I* > 2σ(*I*)
graphite	*R*_int_ = 0.058
phi and ω scans	θ_max_ = 26.0°, θ_min_ = 1.9°
Absorption correction: multi-scan (*SADABS*; Bruker, 2000)	*h* = −26→26
*T*_min_ = 0.646, *T*_max_ = 0.700	*k* = −16→9
8862 measured reflections	*l* = −15→15

### Refinement

**Table:**

Refinement on *F*^2^	Primary atom site location: structure-invariant direct methods
Least-squares matrix: full	Secondary atom site location: difference Fourier map
*R*[*F*^2^ > 2σ(*F*^2^)] = 0.057	Hydrogen site location: inferred from neighbouring sites
*wR*(*F*^2^) = 0.126	H-atom parameters constrained
*S* = 1.26	*w* = 1/[σ^2^(*F*_o_^2^) + (0.0422*P*)^2^ + 21.9067*P*] where *P* = (*F*_o_^2^ + 2*F*_c_^2^)/3
1779 reflections	(Δ/σ)_max_ = 0.001
164 parameters	Δρ_max_ = 0.64 e Å^−^^3^
0 restraints	Δρ_min_ = −0.64 e Å^−^^3^

### Special details

**Table:**

Geometry. All e.s.d.'s (except the e.s.d. in the dihedral angle between two l.s. planes) are estimated using the full covariance matrix. The cell e.s.d.'s are taken into account individually in the estimation of e.s.d.'s in distances, angles and torsion angles; correlations between e.s.d.'s in cell parameters are only used when they are defined by crystal symmetry. An approximate (isotropic) treatment of cell e.s.d.'s is used for estimating e.s.d.'s involving l.s. planes.
Refinement. Refinement of *F*^2^ against ALL reflections. The weighted *R*-factor *wR* and goodness of fit *S* are based on *F*^2^, conventional *R*-factors *R* are based on *F*, with *F* set to zero for negative *F*^2^. The threshold expression of *F*^2^ > σ(*F*^2^) is used only for calculating *R*-factors(gt) *etc*. and is not relevant to the choice of reflections for refinement. *R*-factors based on *F*^2^ are statistically about twice as large as those based on *F*, and *R*- factors based on ALL data will be even larger.

### Fractional atomic coordinates and isotropic or equivalent isotropic displacement parameters (Å^2^)

**Table:**

	*x*	*y*	*z*	*U*_iso_*/*U*_eq_	Occ. (<1)
Cu1	0.41085 (3)	0.14426 (5)	1.06815 (5)	0.0203 (2)	
Mn1	0.2500	0.12380 (9)	0.7500	0.0246 (3)	
C1	0.5000	0.1141 (6)	1.3514 (6)	0.0224 (16)	
H1	0.5000	0.1054	1.4259	0.027*	
C2	0.4501 (2)	0.1221 (4)	1.2832 (4)	0.0191 (11)	
C3	0.3821 (2)	0.1194 (4)	1.2885 (4)	0.0205 (11)	
C4	0.5000	0.1212 (5)	0.7831 (6)	0.0172 (15)	
H4	0.5000	0.1178	0.7081	0.021*	
C5	0.4497 (2)	0.1244 (4)	0.8532 (4)	0.0171 (10)	
C6	0.3809 (2)	0.1249 (4)	0.8491 (4)	0.0183 (10)	
N1	0.47006 (19)	0.1342 (3)	1.1814 (3)	0.0202 (9)	
N2	0.46982 (19)	0.1290 (4)	0.9544 (3)	0.0210 (10)	
O1	0.35447 (17)	0.1124 (3)	1.3755 (3)	0.0298 (10)	
O2	0.35551 (16)	0.1236 (3)	1.1967 (3)	0.0230 (8)	
O3	0.35519 (16)	0.1275 (3)	0.9417 (3)	0.0256 (9)	
O4	0.35220 (16)	0.1236 (3)	0.7623 (3)	0.0229 (8)	
O5W	0.4030 (2)	0.3139 (3)	1.0671 (3)	0.0364 (10)	
H2	0.3857	0.3342	1.1248	0.055*	
H3	0.3823	0.3398	1.0157	0.055*	
O6W	0.2501 (4)	−0.0125 (11)	0.6497 (12)	0.033 (3)	0.50 (3)
H5	0.2677	−0.0653	0.6755	0.049*	0.50 (3)
H6	0.2121	−0.0259	0.6433	0.049*	0.50 (3)
O6W'	0.2644 (7)	0.0351 (14)	0.6051 (12)	0.042 (5)	0.50 (3)
H7	0.2464	0.0563	0.5484	0.063*	0.50 (3)
H8	0.3028	0.0420	0.5955	0.063*	0.50 (3)
O7W	0.2436 (4)	0.2640 (9)	0.8425 (12)	0.032 (3)	0.50 (2)
H9	0.2115	0.2976	0.8258	0.048*	0.50 (2)
H10	0.2752	0.3015	0.8513	0.048*	0.50 (2)
O7W'	0.2415 (5)	0.2143 (15)	0.8986 (13)	0.050 (5)	0.50 (2)
H11	0.2152	0.1755	0.9287	0.076*	0.50 (2)
H12	0.2703	0.1978	0.9416	0.076*	0.50 (2)

### Atomic displacement parameters (Å^2^)

**Table:**

	*U*^11^	*U*^22^	*U*^33^	*U*^12^	*U*^13^	*U*^23^
Cu1	0.0124 (3)	0.0346 (4)	0.0139 (3)	0.0006 (3)	0.0006 (2)	0.0007 (3)
Mn1	0.0162 (5)	0.0320 (7)	0.0255 (6)	0.000	0.0000 (5)	0.000
C1	0.024 (4)	0.024 (4)	0.020 (4)	0.000	0.000	0.004 (3)
C2	0.019 (2)	0.022 (3)	0.016 (2)	0.000 (2)	0.000 (2)	0.000 (2)
C3	0.013 (2)	0.026 (3)	0.023 (3)	−0.006 (2)	0.006 (2)	−0.001 (2)
C4	0.016 (3)	0.021 (4)	0.015 (3)	0.000	0.000	0.000 (3)
C5	0.016 (2)	0.023 (3)	0.013 (2)	−0.004 (2)	−0.0023 (19)	−0.001 (2)
C6	0.018 (2)	0.017 (2)	0.020 (3)	0.002 (2)	−0.004 (2)	0.002 (2)
N1	0.015 (2)	0.030 (3)	0.015 (2)	0.0002 (19)	−0.0011 (16)	0.0025 (18)
N2	0.0120 (19)	0.037 (3)	0.013 (2)	−0.0007 (19)	0.0019 (16)	−0.0018 (19)
O1	0.023 (2)	0.046 (3)	0.021 (2)	−0.0112 (17)	0.0037 (16)	−0.0048 (18)
O2	0.0166 (18)	0.037 (2)	0.0153 (18)	−0.0008 (16)	0.0004 (15)	0.0050 (17)
O3	0.0148 (17)	0.044 (2)	0.018 (2)	−0.0016 (16)	−0.0015 (14)	0.0000 (18)
O4	0.0193 (17)	0.037 (2)	0.0120 (18)	−0.0014 (16)	−0.0030 (14)	0.0009 (16)
O5W	0.050 (3)	0.040 (2)	0.0187 (19)	0.015 (2)	0.0020 (19)	0.0023 (18)
O6W	0.021 (4)	0.040 (6)	0.037 (7)	0.001 (4)	0.003 (4)	0.009 (6)
O6W'	0.038 (6)	0.058 (10)	0.030 (7)	0.022 (7)	−0.016 (5)	−0.007 (7)
O7W	0.018 (4)	0.032 (6)	0.045 (8)	0.004 (4)	−0.004 (4)	−0.001 (6)
O7W'	0.038 (6)	0.072 (11)	0.041 (8)	0.012 (6)	−0.011 (5)	−0.021 (8)

### Geometric parameters (Å, °)

**Table:**

Cu1—N1	1.910 (4)	C3—O2	1.276 (6)
Cu1—N2	1.916 (4)	C4—C5^ii^	1.398 (6)
Cu1—O3	1.992 (4)	C4—C5	1.398 (6)
Cu1—O2	2.014 (4)	C4—H4	0.9300
Cu1—O5W	2.219 (4)	C5—N2	1.329 (6)
Mn1—O6W'	2.157 (10)	C5—C6	1.500 (7)
Mn1—O6W'^i^	2.157 (10)	C6—O4	1.243 (6)
Mn1—O7W	2.162 (9)	C6—O3	1.276 (6)
Mn1—O7W^i^	2.162 (9)	N1—N1^ii^	1.304 (8)
Mn1—O6W	2.168 (10)	N2—N2^ii^	1.315 (8)
Mn1—O6W^i^	2.168 (10)	O5W—H2	0.8500
Mn1—O7W'	2.193 (10)	O5W—H3	0.8500
Mn1—O7W'^i^	2.193 (10)	O6W—H5	0.8500
Mn1—O4	2.231 (3)	O6W—H6	0.8500
Mn1—O4^i^	2.231 (3)	O6W'—H7	0.8500
C1—C2^ii^	1.379 (7)	O6W'—H8	0.8499
C1—C2	1.379 (7)	O7W—H9	0.8500
C1—H1	0.9300	O7W—H10	0.8500
C2—N1	1.342 (6)	O7W'—H11	0.8499
C2—C3	1.484 (7)	O7W'—H12	0.8500
C3—O1	1.238 (6)		
			
N1—Cu1—N2	94.59 (18)	O7W^i^—Mn1—O4^i^	91.7 (2)
N1—Cu1—O3	168.67 (18)	O6W—Mn1—O4^i^	87.8 (3)
N2—Cu1—O3	79.57 (16)	O6W^i^—Mn1—O4^i^	92.1 (3)
N1—Cu1—O2	79.32 (16)	O7W'—Mn1—O4^i^	88.5 (3)
N2—Cu1—O2	165.40 (18)	O7W'^i^—Mn1—O4^i^	91.6 (3)
O3—Cu1—O2	104.02 (14)	O4—Mn1—O4^i^	179.8 (2)
N1—Cu1—O5W	97.17 (17)	C2^ii^—C1—C2	103.9 (7)
N2—Cu1—O5W	98.69 (18)	C2^ii^—C1—H1	128.1
O3—Cu1—O5W	93.33 (16)	C2—C1—H1	128.1
O2—Cu1—O5W	95.25 (15)	N1—C2—C1	109.2 (5)
O6W'—Mn1—O6W'^i^	115.2 (12)	N1—C2—C3	111.5 (4)
O6W'—Mn1—O7W	154.6 (8)	C1—C2—C3	139.3 (5)
O6W'^i^—Mn1—O7W	90.2 (7)	O1—C3—O2	124.0 (5)
O6W'—Mn1—O7W^i^	90.2 (7)	O1—C3—C2	121.7 (5)
O6W'^i^—Mn1—O7W^i^	154.6 (8)	O2—C3—C2	114.4 (4)
O7W—Mn1—O7W^i^	64.5 (9)	C5^ii^—C4—C5	103.2 (6)
O6W'—Mn1—O6W	23.7 (3)	C5^ii^—C4—H4	128.4
O6W'^i^—Mn1—O6W	92.1 (11)	C5—C4—H4	128.4
O7W—Mn1—O6W	175.4 (5)	N2—C5—C4	109.2 (4)
O7W^i^—Mn1—O6W	112.9 (6)	N2—C5—C6	111.2 (4)
O6W'—Mn1—O6W^i^	92.1 (11)	C4—C5—C6	139.6 (5)
O6W'^i^—Mn1—O6W^i^	23.7 (3)	O4—C6—O3	123.8 (5)
O7W—Mn1—O6W^i^	112.9 (6)	O4—C6—C5	122.1 (5)
O7W^i^—Mn1—O6W^i^	175.4 (5)	O3—C6—C5	114.0 (4)
O6W—Mn1—O6W^i^	69.9 (10)	N1^ii^—N1—C2	108.9 (3)
O6W'—Mn1—O7W'	176.5 (5)	N1^ii^—N1—Cu1	132.47 (12)
O6W'^i^—Mn1—O7W'	65.1 (8)	C2—N1—Cu1	118.6 (3)
O7W—Mn1—O7W'	25.2 (3)	N2^ii^—N2—C5	109.3 (3)
O7W^i^—Mn1—O7W'	89.7 (10)	N2^ii^—N2—Cu1	132.09 (12)
O6W—Mn1—O7W'	157.2 (8)	C5—N2—Cu1	118.5 (3)
O6W^i^—Mn1—O7W'	87.7 (8)	C3—O2—Cu1	116.0 (3)
O6W'—Mn1—O7W'^i^	65.1 (8)	C6—O3—Cu1	116.3 (3)
O6W'^i^—Mn1—O7W'^i^	176.5 (5)	C6—O4—Mn1	124.1 (3)
O7W—Mn1—O7W'^i^	89.7 (10)	Cu1—O5W—H2	109.9
O7W^i^—Mn1—O7W'^i^	25.2 (3)	Cu1—O5W—H3	116.2
O6W—Mn1—O7W'^i^	87.7 (8)	H2—O5W—H3	105.7
O6W^i^—Mn1—O7W'^i^	157.2 (8)	Mn1—O6W—H5	116.8
O7W'—Mn1—O7W'^i^	114.9 (13)	Mn1—O6W—H6	102.7
O6W'—Mn1—O4	84.9 (4)	H5—O6W—H6	107.7
O6W'^i^—Mn1—O4	95.0 (4)	Mn1—O6W'—H6	84.8
O7W—Mn1—O4	91.7 (2)	Mn1—O6W'—H7	116.4
O7W^i^—Mn1—O4	88.5 (2)	H6—O6W'—H7	94.9
O6W—Mn1—O4	92.1 (3)	Mn1—O7W—H9	111.1
O6W^i^—Mn1—O4	87.8 (3)	Mn1—O7W—H10	120.1
O7W'—Mn1—O4	91.6 (3)	H9—O7W—H10	113.6
O7W'^i^—Mn1—O4	88.5 (3)	Mn1—O7W'—H11	96.0
O6W'—Mn1—O4^i^	95.0 (4)	Mn1—O7W'—H12	109.1
O6W'^i^—Mn1—O4^i^	84.9 (4)	H11—O7W'—H12	94.1
O7W—Mn1—O4^i^	88.5 (2)		

Symmetry codes: (i) −*x*+1/2, *y*, −*z*+3/2; (ii) −*x*+1, *y*, *z*.
